# β-Lactamase Genes without Limits

**DOI:** 10.3390/microorganisms11051200

**Published:** 2023-05-04

**Authors:** Thierry Naas, Laura Dabos, Rémy A. Bonnin

**Affiliations:** 1 Team ReSIST, INSERM U1184, Université Paris Saclay, CEA, Inserm, Immunologie des Maladies Virales, Auto-Immunes, Hématologiques et Bactériennes (IMVA-HB/IDMIT), 92265 Fontenay-Aux-Roses & Kremlin Bicêtre, Franceremy.bonnin@u-psud.fr (R.A.B.); 2 Service de Bactériologie-Hygiène, Hôpital Bicêtre, AP-HP Paris-Saclay, 94270 Le Kremlin-Bicêtre, France; 3 French National Reference Center for Carbapenem-Resistant Enterobacterales, Hôpital Bicêtre, 94270 Le Kremlin-Bicêtre, France; 4 Centro de Biotecnología y Genómica de Plantas (UPM-INIA) Vía de Servicio M-40 (Campus de Montegancedo) KM 38, 28223 Pozuelo de Alarcón, Spain

β-Lactams are among the most prescribed antibiotics worldwide, mainly due to their weak toxicity and good efficacy. However, their clinical use is currently threatened by the worldwide spread of β-lactamases (BLs) capable of hydrolyzing them, especially among multidrug-resistant (MDR) Gram-negative bacteria (GNB). With the increase in the incidence of GNB infections for which few effective treatments exist, the contribution of drug-hydrolyzing enzymes, most notably β-lactamases, to this serious clinical problem also increases [1]. Currently, β-lactamase-mediated resistance does not spare even the newest and most powerful β-lactams, the activity of which is challenged by class B metallo-β-lactamases (MBLs) (e.g., IMP, VIM, and NDM) and classes A and D serine-carbapenemases (e.g., KPC, IMI, GES, OXA-48, OXA-23, and OXA-40) [1,2,3]. The number of clinically relevant β-lactamases that have been identified has drastically increased in recent years [4]. They correspond to the point mutant derivatives of either known or novel enzymes [3]. This large heterogeneity of enzymes illustrates the formidable potential of bacteria to adapt themselves to hostile environments and fight against one of the main antibiotic families [4,5,6]. This Special Issue involves all the aspects related to β-lactamase research, with special emphasis on the different health areas (human, veterinarian, and environmental health) of antimicrobial resistance (AMR). Thus, 12 highly topical articles were selected for this Special Issue.

Long-term care facilities have been revealed as reservoirs of MDR bacteria. Piccirilli et al. investigated the molecular mechanism sustaining the carbapenem resistance of 68 carbapenem-resistant *K. pneumoniae* collected from 12 long-term care facilities (LTCFs) in northern Italy [7]. WGS analysis showed that ST307, ST512, and ST37 were the main lineages diffused among the LTCFs expressing either *bla*_KPC-2_, *bla*_KPC-3_, *bla*_KPC-9_, or *bla*_OXA-23_ carbapenemase genes. The plasmid-encoded *bla*_OXA-23_ gene, together with *bla*_CTX-M-15_, *bla*_OXA-1_, and *bla*_LEN-12_ genes, were identified in two ST512 isolated from two residents of the same LTCF, underlining further spread of the *bla*_OXA-23_ gene beyond *Acinetobacter* spp. and *Proteus mirabilis*. This study highlights the spread of different *K. pneumoniae* lineages in LTCFs in Italy, which may be related not only to cross-transmission between patients but also to contaminated surfaces in patients’ rooms. The ability to reduce the digestive carriage with these MDR bacteria will reduce the spread of these bacteria. Javaudin et al. investigated the ability of phage therapy to reduce the digestive carriage of MDR bacteria. Four novel lytic phages were isolated in vitro to assess their efficacy against an ESBL- and OXA-48 carbapenemase-producing *E. coli* strain [8]. The efficacy of oral and rectal phage therapy to reduce ESBL-OXA-48 *E. coli* digestive carriage was tested in a mouse model. This study shows that oral treatment with amoxicillin promotes digestive carriage in mice, and it also confirms the difficulty of achieving efficacy with phage therapy to reduce MDR bacterial digestive carriage in vivo. Alternatively, decontamination of patients’ environment is crucial to reduce the spread of these bacteria, but this requires effective tools to monitor environmental decontamination. Stein et al. presented an integrative method using polywipes (buffered wipes for sampling large areas), to detect CPE on surfaces/fomites in the patient environment [9]. They examined environmental samples from 28 rooms occupied with CPE-colonized patients, as revealed using CPE rectal screening. With culture- and molecular-based approaches, cleaning and disinfection strategies were more effectively implemented in everyday clinical practice. The positive detection rates fluctuated between 30.5% (the mean percentage of positive results after 72 h) and 35.2% (after 24 h and one week). These results confirm the role of patient-side contamination in nosocomial transmission and highlight the decisive role of appropriate decontamination strategies for the effective control of infection.

As β-lactamase genes spread, they may acquire point mutations that result in changes in their hydrolysis profile. This is the case with TEM β-lactamases, with more than 245 recognized variants [4]. Madzgalla et al. aimed to evaluate the role of specific substitutions on the resistance phenotype of TEM variants, identify new phenotype-relevant amino acid substitutions (PRASs), and investigate the effectiveness of mathematical prediction models [10]. PRASs (i) were predicted using mathematical approximation algorithms based on the NCBI National Database of Antibiotic Resistant Organisms and (ii) were then experimentally confirmed. Mathematical models accurately predicted the strongest phenotype-relevant substitutions but presented challenges in identifying less prevalent but still phenotype-modifying ones. OXA-48 is another example of a worldwide spreading β-lactamase [11] that evolved through mutation, leading to major differences in their hydrolytic profile [2,4,11]. In the study by Dabos et al., a hydrolytic cutoff was established to determine when an OXA-48-like enzyme may be considered a carbapenemase [12]. The coefficient of activity for imipenem (kcat/Km), determined for a total of 30 OXA-48-like variants, decreased from 550 mM^−1^.s^−1^ to 0.02 mM^−1^.s^−1^. To match the kcat/Km results with biochemical validation tests, Dabos et al. defined the value of 0.27 mM-1.s-1 as the cutoff above which an OXA-48 variant may be considered a carbapenem-hydrolyzing enzyme. This diversity of OXA-48 variants in terms of hydrolysis profile and amino acid sequences may also have an impact on detection with commercially available assays (either biochemical or molecular) and thus contribute to the silent spread of some variants, as observed in OXA-244 [13] and OXA-900, a novel and distinct subfamily of OXA-48 variants described by Frenk et al. [14]. The IncC plasmid-mediated *bla*_OXA-900_ gene was identified in a *Citrobacter freundii* isolate that displayed significant carbapenem hydrolysis using β-Carba (BioRad, Marnes-la-Coquette, France) and a modified CIM test, but it was not detected using commercially available DNA-based methods. The *bla*_OXA-900_ gene was located on an antibiotic resistance island (ARI), together with two ESBL genes (*bla*_CTX-M-39_ and *bla*_PER-2_ genes).

ESBL-producing isolates have been extensively investigated around the world, in both hospital and community settings. Carvalho et al. studied 62 cefotaxime-/ceftazidime-resistant *E. coli* (*n* = 38) and *K. pneumoniae* (*n* = 24) clinical isolates from Portugal [15]. ESBL activity was detected in all *E. coli* and *K. pneumoniae* isolates. In this study, different CTX-M variants (mainly CTX-M-15) and SHV-type variants (mainly SHV-12) were detected. As infections with ESBL producers are difficult to treat, they may lead to relapsing infections. Karima et al. studied 123 cases of recurrent urinary tract infections (RUTIs) to investigate whether they were due to the same or a different *E. coli* isolate. In only five events, changes were detected in the ESBL *E. coli* strain between RUTI episodes. The transmission of *bla*_CTX-M_ plasmids seemed to be a rare event during RUTIs [16]. Finally, Gaviria et al. investigated antimicrobial susceptibility, antimicrobial resistance genes, and the biological diversity of urinary ESBL-producing *E. coli* isolates at Cerdanya Hospital, a European cross-border (between France and Spain) hospital [17]. All the studied isolates were multidrug-resistant and belonged to 19 different sequence types showing high genetic diversity. The most prevalent ESBL enzymes were CTX-M-14 and CTX-M-15. The results provide evidence supporting the absence of a single predominant clone of ESBL-MDR-*E. coli* at Cerdanya Hospital. ESBL-producing isolates have now been investigated in all areas of animal health, including companion, livestock, and wild animals. In the study of Witte et al., the authors presented evidence of the fecal carriage of broad-spectrum β-lactamase-producing Enterobacterales in zoo mammals in two Belgian zoos, likely reflecting the fact that exotic animals are brought in proximity of humans [18]. Thirty-five ceftiofur-resistant isolates were obtained from 52.6% of zoo mammals. Most isolates were identified as *E. coli* (25/35), 64.0% of which showed multidrug resistance (MDR). The main β-lactamases were CTX-M-1 (17/25) and TEM-1 (4/25). In the case of livestock animals, MDR bacteria may enter the food chain and thus be responsible for AMR spread. Abboud et al. sought to investigate the etiology of the main mastitis-causing pathogens in northern Lebanon, determine their antimicrobial susceptibility profiles, and identify their antimicrobial resistance (AMR) genes [19]. A total of 101 quarter-milk samples were collected from 77 cows and 11 goats presenting symptoms of mastitis on 45 dairy farms. The most frequently identified species were *Streptococcus uberis* (19.2%), *Streptococcus agalactiae* (15.1%), *E. coli* (12.3%), and *Staphylococcus aureus* (10.96%). This study reveals the presence of *bla*_ESBL_ and *mecA* genes in the Lebanese dairy chain and underlines the importance of monitoring the consequences of antimicrobial usage in animals. Similarly, Carey et al. investigated plasmid-mediated fluoroquinolone and macrolide resistance genes among ESBL-producing *E. coli* isolates from 147 dairy calves [20]. The *qnrB*, *mph(A)*, and group 1 *bla*_CTX-M_ genes were identified with higher abundance in young calves.

## Conclusions

This Special Issue provides bodies of evidence highlighting the spread of ESBL and carbapenemases in all elements of a one-health setting (human, animal, and environment). All the manuscripts amassed in this collection intersect in pointing to the spread of broad-spectrum β-lactamases worldwide. The scientific community currently faces an unresolved challenge in how to reverse the spread of these resistance genes often identified in highly successful clones.

## Data Availability

Not acceptable.
